# Tobacco

**Published:** 1872-03

**Authors:** 


### Tobacco.

In the November part of the *Royal London Ophthalmic Hospital Reports*, Mr. Jonathan Hutchison gives an account of his further experience in respect to amaurosis supposed to be due to tobacco. It will be remembered that he has previously written on the subject, his first paper appearing in the *London Hospital Reports* for 1864; his second in the *Medico-Chirurgical Transactions* for 1867. He tells us that "idiopathic amaurosis" appears in great disproportion between the two sexes. In his first series the numbers were thirty-seven men and three women; in the second thirty-four men and five women; in this third series we find twenty-eight men and only one woman. We should be very glad to learn from any of our foreign correspondents in countries where both sexes smoke, whether the same disproportion of cases of amaurosis is observed in men and women. Mr. Hutchinson has carefully investigated other possible causes, and still believes that tobacco is the real one. He notices thot most of the sufferers smoked shag, and pronounces that "the most deleterious form of tobacco." But we take leave to remind him that most of his cases are hospital ones—the patients therefore poor—and shag is the *cheapest* form of tobacco. Morover, it is perhaps the most commonly used form, even among those who can afford a more expensive quality. Mr. Hutchison has found in the early stages that the vision improves when the disuse of tobacco is real and complete, and therefore considers it a duty to urge complete, immediate abstinence.—*Cincinnati Lancet and Observer.*

